# 1-[4-(4-Chloro­but­oxy)-2-hy­droxy­phen­yl]ethanone

**DOI:** 10.1107/S1600536811002169

**Published:** 2011-01-22

**Authors:** Li Wang, Jun-Peng Zhan, Jian-Qiang Liang, Zhe-Fei Li, Ge-Hong Wei

**Affiliations:** a College of Life Sciences, Northwest A&F University, Yangling Shaanxi 712100, People’s Republic of China

## Abstract

In the title compound, C_12_H_15_ClO_3_, the eth­oxy group is nearly coplanar with the benzene ring, making a dihedral angle of 9.03 (4)°, and is involved in an intra­molecular O—H⋯O hydrogen bond to the neighbouring hy­droxy group.

## Related literature

For the synthesis of the title comppund, see: Dermer (1934 ▶). For related structures, see: Schlemper (1986 ▶).
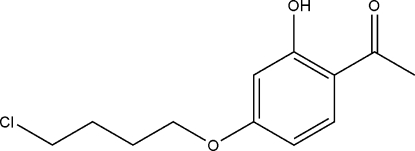


## Experimental

### 

#### Crystal data

C_12_H_15_ClO_3_
*M* *_r_* = 242.69
Triclinic, 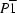

*a* = 5.2750 (4) Å
*b* = 9.8941 (10) Å
*c* = 11.6529 (12) Å
α = 99.735 (2)°
β = 98.242 (1)°
γ = 92.248 (1)°
*V* = 591.97 (10) Å^3^
*Z* = 2
Mo *K*α radiation
μ = 0.31 mm^−1^
*T* = 298 K
0.49 × 0.40 × 0.24 mm

#### Data collection

Bruker SMART CCD area-detector diffractometer
Absorption correction: multi-scan (*SADABS*; Sheldrick, 1996 ▶) *T*_min_ = 0.862, *T*_max_ = 0.929
3097 measured reflections
2068 independent reflections
1517 reflections with *I* > 2σ(*I*)
*R*_int_ = 0.019

#### Refinement

*R*[*F* ^2^ > 2σ(*F* ^2^)] = 0.040
*wR*(*F* ^2^) = 0.108
*S* = 1.06
2068 reflections
147 parameters
H-atom parameters constrained
Δρ_max_ = 0.20 e Å^−3^
Δρ_min_ = −0.21 e Å^−3^

### 

Data collection: *SMART* (Bruker, 1996 ▶); cell refinement: *SAINT* (Bruker, 1996 ▶); data reduction: *SAINT*; program(s) used to solve structure: *SHELXS97* (Sheldrick, 2008 ▶); program(s) used to refine structure: *SHELXL97* (Sheldrick, 2008 ▶); molecular graphics: *SHELXTL* (Sheldrick, 2008 ▶); software used to prepare material for publication: *SHELXTL*.

## Supplementary Material

Crystal structure: contains datablocks I, global. DOI: 10.1107/S1600536811002169/nc2215sup1.cif

Structure factors: contains datablocks I. DOI: 10.1107/S1600536811002169/nc2215Isup2.hkl

Additional supplementary materials:  crystallographic information; 3D view; checkCIF report

## Figures and Tables

**Table 1:** Hydrogen-bond geometry (Å, °)

*D*—H⋯*A*	*D*—H	H⋯*A*	*D*⋯*A*	*D*—H⋯*A*
O2—H2⋯O1	0.82	1.82	2.539 (2)	146

**Fig. 1.**
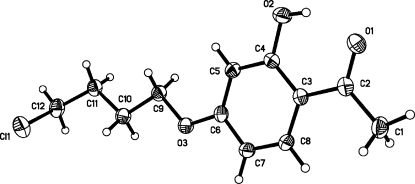
Crystal structure of the title compound with labeling and displacement ellipsoids drawn at the 30% probability level.

## supplementary crystallographic information

### Comment

The crystal structure of the title compound was determined as a part of a project on the synthesis of new acetophenone derivatives. To clearly identify the product a single crystal X-ray analysis was performed.

In the crystal structure of the title compound the dihedral angle between the benzene ring C3—C8 and the ethoxy group is (O3, C9 and C10) amount to 9.03 (4)°. The carbonyl oxygen atom is involved in intramolecular O—H···O hydrogen bonding to the neighbored hydroxy group

### Experimental

2, 4-Dihydroxyacetonephenone (5 mmol), potassium carbonate (6 mmol), 1-bromo-4-chlorobutane (5 mmol) and 50 ml acetone were mixed in a 100 ml flask. After 2.5 h stirring at 329 K the crude product was filtered off. Single crystals suitable for X-ray analysis were obtained by slow evaporation of the solvent from a solution of the title compound in n-hexane/ethyl acetate/methanol (3:3:1, V/V)at room temperature.

### Refinement

The H atoms were positioned with idealized geometry (O-H H atoms allowed to rotate but no to tip) with C—H distance in the range of 0.93–0.97 Å and O—H distance of 0.82 Å, and refined as riding, with *U*_iso_(H)= 1.2–1.5*U*_eq_(C,*O*).

### Crystal data

**Table:**

C_12_H_15_ClO_3_	*F*(000) = 256
*M**_r_* = 242.69	*D*_x_ = 1.362 Mg m^−^^3^
Triclinic, *P*1	Melting point = 317–318 K
*a* = 5.2750 (4) Å	Mo *K*α radiation, λ = 0.71073 Å
*b* = 9.8941 (10) Å	Cell parameters from 1252 reflections
*c* = 11.6529 (12) Å	θ = 2.5–27.5°
α = 99.735 (2)°	µ = 0.31 mm^−^^1^
β = 98.242 (1)°	*T* = 298 K
γ = 92.248 (1)°	Triclinic, colourless
*V* = 591.97 (10) Å^3^	0.49 × 0.40 × 0.24 mm
*Z* = 2	

### Data collection

**Table:**

Bruker SMART CCD area-detector diffractometer	2068 independent reflections
Radiation source: fine-focus sealed tube	1517 reflections with *I* > 2σ(*I*)
graphite	*R*_int_ = 0.019
φ and ω scans	θ_max_ = 25.0°, θ_min_ = 2.5°
Absorption correction: multi-scan (*SADABS*; Sheldrick, 1996)	*h* = −6→5
*T*_min_ = 0.862, *T*_max_ = 0.929	*k* = −11→9
3097 measured reflections	*l* = −13→13

### Refinement

**Table:**

Refinement on *F*^2^	Secondary atom site location: difference Fourier map
Least-squares matrix: full	Hydrogen site location: inferred from neighbouring sites
*R*[*F*^2^ > 2σ(*F*^2^)] = 0.040	H-atom parameters constrained
*wR*(*F*^2^) = 0.108	*w* = 1/[σ^2^(*F*_o_^2^) + (0.0463*P*)^2^ + 0.1542*P*] where *P* = (*F*_o_^2^ + 2*F*_c_^2^)/3
*S* = 1.06	(Δ/σ)_max_ < 0.001
2068 reflections	Δρ_max_ = 0.20 e Å^−^^3^
147 parameters	Δρ_min_ = −0.21 e Å^−^^3^
0 restraints	Extinction correction: *SHELXL97* (Sheldrick, 2008), Fc^*^=kFc[1+0.001xFc^2^λ^3^/sin(2θ)]^-1/4^
Primary atom site location: structure-invariant direct methods	Extinction coefficient: 0.294 (14)

### Special details

**Table:**

Geometry. All e.s.d.'s (except the e.s.d. in the dihedral angle between two l.s. planes) are estimated using the full covariance matrix. The cell e.s.d.'s are taken into account individually in the estimation of e.s.d.'s in distances, angles and torsion angles; correlations between e.s.d.'s in cell parameters are only used when they are defined by crystal symmetry. An approximate (isotropic) treatment of cell e.s.d.'s is used for estimating e.s.d.'s involving l.s. planes.
Refinement. Refinement of *F*^2^ against ALL reflections. The weighted *R*-factor *wR* and goodness of fit *S* are based on *F*^2^, conventional *R*-factors *R* are based on *F*, with *F* set to zero for negative *F*^2^. The threshold expression of *F*^2^ > σ(*F*^2^) is used only for calculating *R*-factors(gt) *etc*. and is not relevant to the choice of reflections for refinement. *R*-factors based on *F*^2^ are statistically about twice as large as those based on *F*, and *R*- factors based on ALL data will be even larger.

### Fractional atomic coordinates and isotropic or equivalent isotropic displacement parameters (Å^2^)

**Table:**

	*x*	*y*	*z*	*U*_iso_*/*U*_eq_	
Cl1	0.69127 (14)	0.39709 (8)	0.91715 (5)	0.0668 (3)	
O1	−0.6516 (3)	−0.02779 (17)	0.10226 (14)	0.0545 (5)	
O2	−0.2483 (3)	−0.06891 (15)	0.23591 (14)	0.0513 (5)	
H2	−0.3821	−0.0896	0.1897	0.077*	
O3	0.2113 (3)	0.32392 (15)	0.48072 (12)	0.0444 (4)	
C1	−0.7899 (4)	0.1907 (3)	0.0771 (2)	0.0508 (6)	
H1A	−0.9234	0.1367	0.0217	0.076*	
H1B	−0.6955	0.2480	0.0370	0.076*	
H1C	−0.8647	0.2473	0.1371	0.076*	
C2	−0.6130 (4)	0.0977 (2)	0.13236 (17)	0.0385 (5)	
C3	−0.3944 (4)	0.1550 (2)	0.22183 (17)	0.0338 (5)	
C4	−0.2210 (4)	0.0684 (2)	0.27035 (17)	0.0349 (5)	
C5	−0.0135 (4)	0.1209 (2)	0.35608 (18)	0.0379 (5)	
H5	0.1012	0.0623	0.3866	0.045*	
C6	0.0206 (4)	0.2611 (2)	0.39548 (17)	0.0357 (5)	
C7	−0.1489 (4)	0.3496 (2)	0.34910 (18)	0.0407 (5)	
H7	−0.1254	0.4439	0.3761	0.049*	
C8	−0.3499 (4)	0.2967 (2)	0.26358 (18)	0.0390 (5)	
H8	−0.4606	0.3566	0.2321	0.047*	
C9	0.3983 (4)	0.2437 (2)	0.53460 (18)	0.0400 (5)	
H9A	0.5055	0.2040	0.4783	0.048*	
H9B	0.3150	0.1700	0.5636	0.048*	
C10	0.5562 (4)	0.3405 (2)	0.63445 (19)	0.0433 (6)	
H10A	0.4454	0.3780	0.6899	0.052*	
H10B	0.6285	0.4163	0.6039	0.052*	
C11	0.7719 (4)	0.2729 (2)	0.69890 (18)	0.0440 (6)	
H11A	0.7030	0.1880	0.7170	0.053*	
H11B	0.8976	0.2495	0.6469	0.053*	
C12	0.9052 (4)	0.3603 (3)	0.8113 (2)	0.0517 (6)	
H12A	0.9737	0.4459	0.7941	0.062*	
H12B	1.0479	0.3128	0.8443	0.062*	

### Atomic displacement parameters (Å^2^)

**Table:**

	*U*^11^	*U*^22^	*U*^33^	*U*^12^	*U*^13^	*U*^23^
Cl1	0.0704 (5)	0.0785 (5)	0.0487 (4)	0.0060 (4)	0.0128 (3)	0.0000 (3)
O1	0.0550 (10)	0.0450 (10)	0.0549 (10)	−0.0085 (8)	−0.0092 (8)	0.0018 (8)
O2	0.0548 (10)	0.0345 (9)	0.0583 (10)	0.0009 (7)	−0.0059 (8)	0.0026 (7)
O3	0.0404 (9)	0.0406 (9)	0.0452 (9)	0.0034 (7)	−0.0114 (7)	0.0026 (7)
C1	0.0449 (14)	0.0580 (16)	0.0450 (13)	−0.0012 (11)	−0.0079 (11)	0.0097 (11)
C2	0.0355 (12)	0.0454 (14)	0.0339 (11)	−0.0028 (10)	0.0052 (9)	0.0068 (10)
C3	0.0321 (11)	0.0373 (12)	0.0317 (10)	0.0002 (9)	0.0054 (9)	0.0048 (9)
C4	0.0366 (12)	0.0329 (12)	0.0346 (11)	0.0012 (9)	0.0068 (9)	0.0031 (9)
C5	0.0367 (12)	0.0390 (13)	0.0382 (11)	0.0078 (9)	0.0028 (9)	0.0086 (9)
C6	0.0321 (11)	0.0416 (13)	0.0326 (10)	0.0005 (9)	0.0027 (9)	0.0062 (9)
C7	0.0428 (13)	0.0336 (12)	0.0426 (12)	0.0037 (9)	−0.0018 (10)	0.0042 (10)
C8	0.0379 (12)	0.0394 (13)	0.0384 (11)	0.0065 (9)	−0.0019 (9)	0.0087 (9)
C9	0.0368 (12)	0.0439 (13)	0.0390 (12)	0.0068 (10)	0.0033 (9)	0.0074 (10)
C10	0.0390 (12)	0.0444 (13)	0.0434 (12)	0.0035 (10)	−0.0021 (10)	0.0057 (10)
C11	0.0360 (12)	0.0546 (15)	0.0408 (12)	0.0086 (10)	0.0033 (10)	0.0079 (11)
C12	0.0396 (13)	0.0678 (17)	0.0460 (13)	0.0051 (11)	−0.0020 (11)	0.0123 (12)

### Geometric parameters (Å, °)

**Table:**

Cl1—C12	1.791 (2)	C6—C7	1.393 (3)
O1—C2	1.232 (2)	C7—C8	1.367 (3)
O2—C4	1.346 (2)	C7—H7	0.9300
O2—H2	0.8200	C8—H8	0.9300
O3—C6	1.357 (2)	C9—C10	1.499 (3)
O3—C9	1.431 (2)	C9—H9A	0.9700
C1—C2	1.493 (3)	C9—H9B	0.9700
C1—H1A	0.9600	C10—C11	1.513 (3)
C1—H1B	0.9600	C10—H10A	0.9700
C1—H1C	0.9600	C10—H10B	0.9700
C2—C3	1.464 (3)	C11—C12	1.504 (3)
C3—C4	1.402 (3)	C11—H11A	0.9700
C3—C8	1.403 (3)	C11—H11B	0.9700
C4—C5	1.390 (3)	C12—H12A	0.9700
C5—C6	1.382 (3)	C12—H12B	0.9700
C5—H5	0.9300		
			
C4—O2—H2	109.5	C7—C8—H8	119.0
C6—O3—C9	119.86 (16)	C3—C8—H8	119.0
C2—C1—H1A	109.5	O3—C9—C10	106.13 (17)
C2—C1—H1B	109.5	O3—C9—H9A	110.5
H1A—C1—H1B	109.5	C10—C9—H9A	110.5
C2—C1—H1C	109.5	O3—C9—H9B	110.5
H1A—C1—H1C	109.5	C10—C9—H9B	110.5
H1B—C1—H1C	109.5	H9A—C9—H9B	108.7
O1—C2—C3	120.04 (19)	C9—C10—C11	113.12 (18)
O1—C2—C1	119.69 (19)	C9—C10—H10A	109.0
C3—C2—C1	120.26 (19)	C11—C10—H10A	109.0
C4—C3—C8	117.24 (18)	C9—C10—H10B	109.0
C4—C3—C2	120.54 (18)	C11—C10—H10B	109.0
C8—C3—C2	122.21 (18)	H10A—C10—H10B	107.8
O2—C4—C5	117.17 (18)	C12—C11—C10	114.25 (19)
O2—C4—C3	121.47 (18)	C12—C11—H11A	108.7
C5—C4—C3	121.36 (19)	C10—C11—H11A	108.7
C6—C5—C4	119.31 (19)	C12—C11—H11B	108.7
C6—C5—H5	120.3	C10—C11—H11B	108.7
C4—C5—H5	120.3	H11A—C11—H11B	107.6
O3—C6—C5	124.69 (18)	C11—C12—Cl1	111.58 (16)
O3—C6—C7	114.72 (18)	C11—C12—H12A	109.3
C5—C6—C7	120.59 (19)	Cl1—C12—H12A	109.3
C8—C7—C6	119.5 (2)	C11—C12—H12B	109.3
C8—C7—H7	120.3	Cl1—C12—H12B	109.3
C6—C7—H7	120.3	H12A—C12—H12B	108.0
C7—C8—C3	122.01 (19)		
			
O1—C2—C3—C4	2.8 (3)	C4—C5—C6—O3	178.18 (18)
C1—C2—C3—C4	−177.19 (19)	C4—C5—C6—C7	−0.8 (3)
O1—C2—C3—C8	−176.56 (19)	O3—C6—C7—C8	−179.25 (18)
C1—C2—C3—C8	3.5 (3)	C5—C6—C7—C8	−0.2 (3)
C8—C3—C4—O2	179.90 (18)	C6—C7—C8—C3	1.1 (3)
C2—C3—C4—O2	0.6 (3)	C4—C3—C8—C7	−0.9 (3)
C8—C3—C4—C5	−0.1 (3)	C2—C3—C8—C7	178.46 (19)
C2—C3—C4—C5	−179.47 (18)	C6—O3—C9—C10	−172.47 (17)
O2—C4—C5—C6	−179.10 (18)	O3—C9—C10—C11	−177.85 (18)
C3—C4—C5—C6	0.9 (3)	C9—C10—C11—C12	−170.21 (19)
C9—O3—C6—C5	1.4 (3)	C10—C11—C12—Cl1	63.0 (2)
C9—O3—C6—C7	−179.63 (17)		

### Hydrogen-bond geometry (Å, °)

**Table:**

*D*—H···*A*	*D*—H	H···*A*	*D*···*A*	*D*—H···*A*
O2—H2···O1	0.82	1.82	2.539 (2)	146
